# Quantum annealing-based clustering of single cell RNA-seq data

**DOI:** 10.1093/bib/bbad377

**Published:** 2023-10-24

**Authors:** Michal Kubacki, Mahesan Niranjan

**Affiliations:** Faculty of Engineering and Physical Sciences, University of Southampton; Faculty of Engineering and Physical Sciences, University of Southampton

**Keywords:** scRNA-seq, quantum annealing, data clustering, combinatorial optimization

## Abstract

Cluster analysis is a crucial stage in the analysis and interpretation of single-cell gene expression (scRNA-seq) data. It is an inherently ill-posed problem whose solutions depend heavily on hyper-parameter and algorithmic choice. The popular approach of K-means clustering, for example, depends heavily on the choice of K and the convergence of the expectation-maximization algorithm to local minima of the objective. Exhaustive search of the space for multiple good quality solutions is known to be a complex problem. Here, we show that quantum computing offers a solution to exploring the cost function of clustering by quantum annealing, implemented on a quantum computing facility offered by D-Wave [1]. Out formulation extracts minimum vertex cover of an affinity graph to sub-sample the cell population and quantum annealing to optimise the cost function. A distribution of low-energy solutions can thus be extracted, offering alternate hypotheses about how genes group together in their space of expressions.

## INTRODUCTION

Data clustering is crucial and especially challenging step in the scRNA-seq analysis workflow [2]. The main challenge stems from the fact that the analysed data are highly dimensional and often represent continuous cell differentiation pathways instead of separate clusters. Generally, we also do not know the exact number of cell types in the sample, and the definition of when to assign a group to a distinct cell identity is not well defined and depends on the type of insight that we look for. Therefore, there does not exist an ideal clustering method because different algorithms will emphasise different aspects of the analysed data. Thus, for example, one of the most popular clustering algorithms, k-means, results in compact and symmetric clusters, aiming to minimise within-cluster variances. Therefore, it is not well suited for the biological data where we expect not symmetric groups of the closely related cells as they progress in time. Also, for the same reason, popular Gaussian mixture models, while more flexible in terms of cluster sizes and shapes, may still perform poorly for single-cell data [3]. On the other hand, the clustering problem can be transformed into the problem of graph partitioning, where we do not assume any particular shape of the partitions. Thus, currently, many of the most popular clustering methodologies are implemented as the graph partitioning problems [2]. Nevertheless, graph partitioning is a representative combinatorial optimization problem that is NP-hard. Therefore, exact solutions quickly become infeasible and we must resort to heuristic algorithms. However, due to the nature of scRNA-seq data, the loss function landscape corresponding to partitioning quality usually contains an abundance of locally optimal solutions representing potential cell classifications. Efficient optimization with such a complex landscape is inherently difficult and naturally leans toward probabilistic techniques such as those based on annealing. In the literature, we can already find examples of simulated annealing applied to the graph partitioning problem [4–6]. However, in this work, we focus exclusively on the quantum annealing implementation, as it has been shown to be a very promising alternative to simulated annealing, capable of outperforming it in terms of both time and solution quality [7–10].

**Figure 0:**
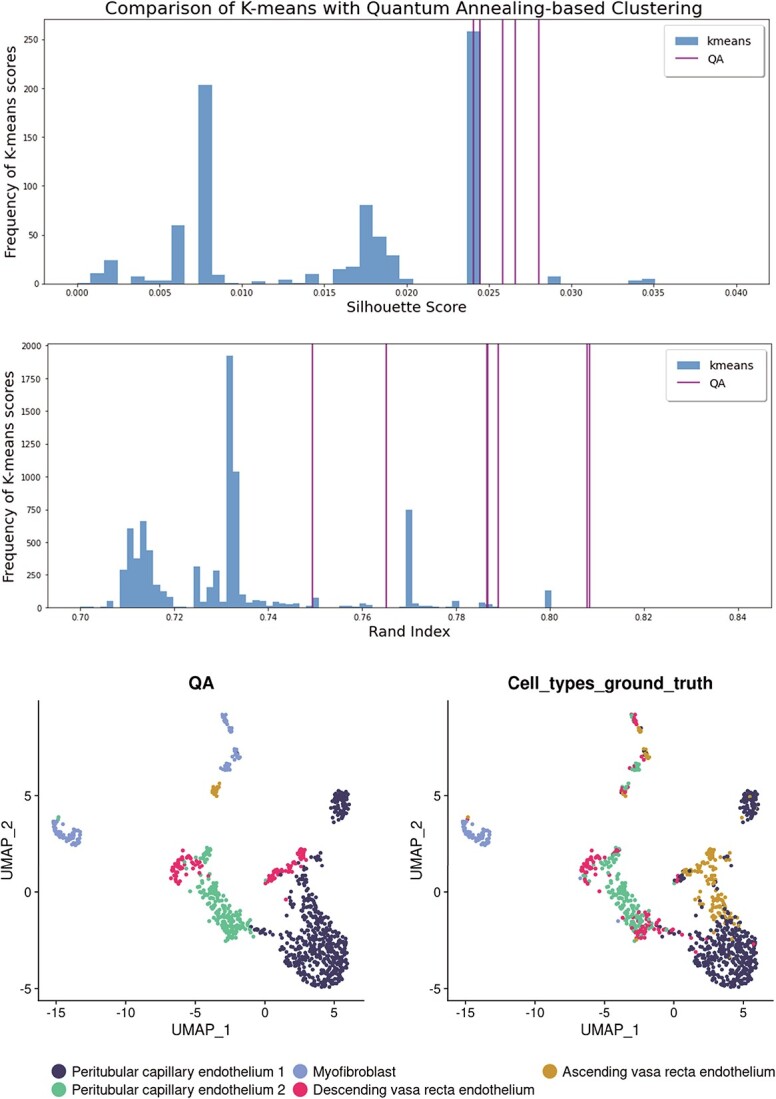
**Comparing K-means and Quantum annealing-based clustering.** Histograms (in blue) show distributions of Silhouette and Rand Index scores [21] for the 9480 different solutions found with the k-means algorithm. On the same graphs, the maroon vertical lines indicate analogous scores of the single solutions sampled from the quantum annealer. It is apparent that the k-means algorithm tends to settle on certain local minima, which can be a limiting factor for data exploration and its conclusions. At the same time, the quantum annealing method samples solution space more holistically and finds solutions that surpass the k-means algorithm in both of the considered metrics. The two graphs at the bottom show the embedding of the dataset [14] using Uniform Manifold Approximation and Projection (UMAP), with the labels corresponding to the dataset annotations (Cell_types_ground_truth) and one of the low-energy solutions found by the quantum annealer (QA). Analysed data includes 1000 cells from the Human-Kidney dataset [14]. Quantum Annealing solutions were obtained using a CQM hybrid solver [19], which is discussed in the supplementary document. An example of the result obtained using K-means clustering is shown in Figure 6.

Although gate model quantum computers of practical use are still a very distant prospect, quantum annealers (QAs, as offered by D-wave company), even nowadays, can be employed to solve real-life problems [11]. They are designed to solve optimization problems and, in this respect, are superior to their gate-model counterparts [12, 13]. In the literature, we can already find examples of successful applications of quantum annealing technology for life-science applications, such as peptides design [14, 15]. Thanks to starting in the equal superposition and quantum tunneling effect, we can much more extensively sample the loss energy function landscape, and so we are more likely to find the global minimum (Due to inherent noise in the quantum systems, common approach to get exact minimum is to at the end of annealing refine found minima with the classical algorithms) or ’difficult to access’ local minima, which the classical simulated annealing would miss. Moreover, after the initial computational cost of problem embedding, with a minimal cost we can tune system parameters and sample a large number of low-energy states. Frequently occurring solutions will correspond to the alternative partitioning, which, as will be discussed later, are invaluable in the case of scRNA-seq analysis.

## RESULTS

Quantum annealing based clustering yields the most significant promise of advantage for the relatively homogeneous clusters. As in those cases, classical algorithms are prone to settle at local minimums and miss more subtle cell partitioning. Accordingly, a subset of the annotated kidney cell dataset [16] was selected, consisting of groups of closely related cell types, making them difficult to examine adequately using classical clustering algorithms. Included manually curated cell types based on gene markers were used as ground truth.

**Figure 1:**
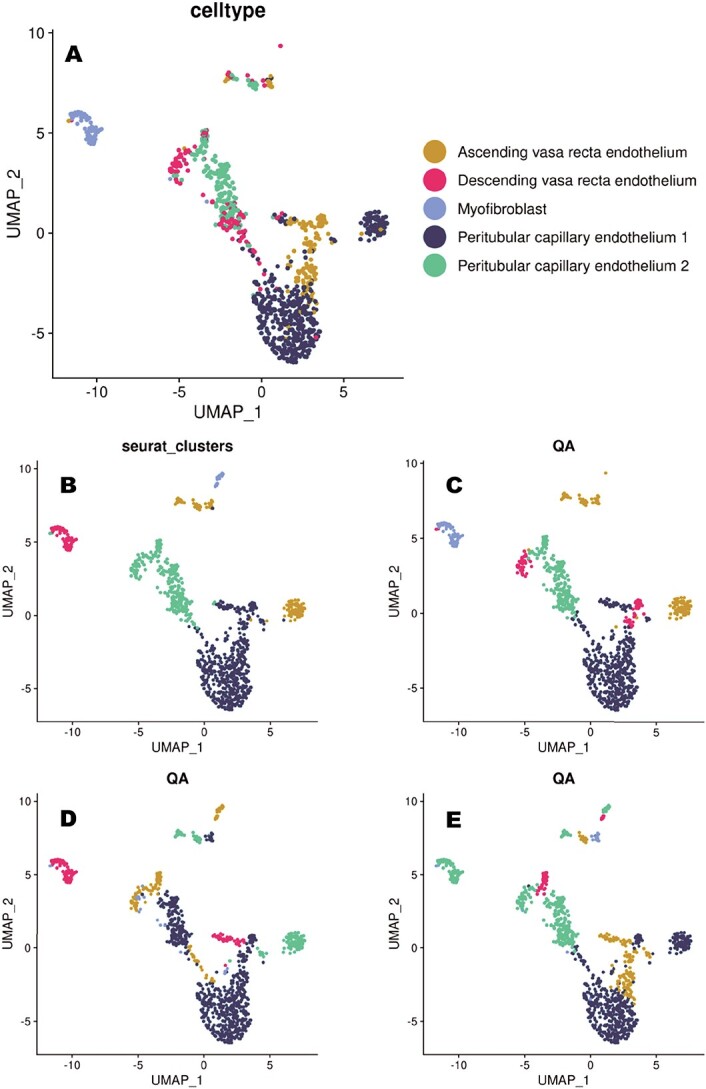
The advantage of quantum annealing in the study of population heterogeneity. (A) shows the original cells’ types annotations. Then in order, Seurat [17] clusters and three of the low-energy states found by the QA are presented. The spectrum of the solutions sampled by the quantum annealer provides better insight into dataset heterogeneity, by correctly suggesting sub-classes of Vasa recta and Peritubular capillary cells. Clusters’ colors in the plots serve only visualisation and do not directly relate to a particular cell type.

Selected subset includes five different cell types: Ascending vasa recta endothelium, Descending vasa recta endothelium, Pertibular capillary endothelium 1, Pertibular capillary endothelium 2 and Myofibroblast. Figure 1 shows the result of the Seurat [17] clustering function and the three consecutive low-energy states sampled by the QA. To some extent, solutions obtained using both methods correctly correlate with the cell identities. However, no single solution indicates the whole heterogeneity of the population.

Thus, for example, the Seurat clustering function fails to distinguish between Peritubular capillary endothelium and Ascending vasa recta endothelium cells. At the same time, one of the lowest energy states presented in the sub-figure *D* found by the annealer does not indicate heterogeneity in the population of Peritubular capillary endothelium cells. In the case of the classical algorithm, to get further insight into data, we need to rerun the whole clustering from the beginning, e.g. with slightly changed clustering parameters or distance metrics. However, in many cases, this will result in very similar, and so not much more informative, data partitioning. The situation is much different for QA, where we can simply check the other low-energy states. For example, as presented in Figure 1, the alternative solutions found by the QA (shown in *E* and *C*) correctly indicate the additional subpopulation missed by the solution in *D*. Thus, joint consideration of the most frequently sampled states of the QA gives us a more accurate insight into the structure of the data set than the classical alternatives.

**Figure 2:**
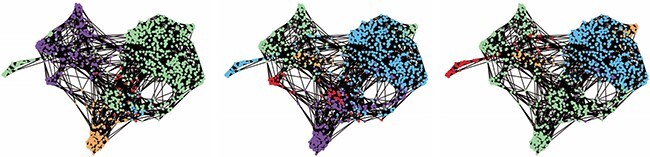
Examples of the first three lowest energy states returned by the QA on 1000 nodes. These are low-energy states of the clustering cost function showing considerable heterogeneity, suggesting multiple useful (low-energy) solutions can be obtained, which will not be the case for classical algorithms like K-means without considerable computing starting from a large number of initial conditions. The colours serve only visualisation and do not directly relate to a particular cell type.

Moreover, as discussed earlier, there may be not something like ideal clustering, so we shouldn’t trust the single clustering result. Instead, biologists would likely benefit from the set of plausible data partitioning (accompanied by the quality scores, indicated by the corresponding energies), which provide deeper insight into the data and supply new promising hypotheses worth additional consideration and testing. In addition, as discussed in the supplementary document, to further explore data, we may emphasise a different aspect of it by tweaking annealing coefficients for each sampling run (this can be done very efficiently, as it doesn’t require a generation of new embedding), imposing minimal expected cluster size and by evaluating solutions confidence (which can be directly related to the sampling distribution). Figure 2 shows the found sets of clusters corresponding to successive low-energy states sampled by the QA, with significant heterogeneity of the found partitions apparent.

The execution time of the clustering procedure described here is not strictly defined, as it depends on the number of states we want to sample from the quantum annealer and the methods for the problem submission, which are discussed in the supplement. However, to give an example, the total execution time for the results shown in Figure 1 is 16 ms spent on the actual quantum annealing and 5 s when we include the time due to the delays of the Web API and the problem embedding procedure.

## METHODOLOGY

This section presents the main idea behind Binary Optimization Model and how it is used for clustering problems and embedded into the QAs. The supplementary document provides more details about actual implementation and available design choices worth consideration. Additionally, helpful information and code examples, including data prepossessing, can be found in GitHub repository and accompanying supplementary document.

### Problem representation

Graph partitioning can be solved through the recursive evaluation of the min-cut problem on the SNN graph. In this graph, nodes represent cells, and edges connect each node to their nearest neighbors with weights corresponding to the Jaccard similarity coefficient between a given pair of cells [17, 18]: 


\begin{align*}& \textit{Jaccard Index between each pair of cells:}\ \ J(A,B)=\frac{\left | A \cap B \right |}{\left | A \cup B \right |} \end{align*}

*where A and B represent the binary differential expression values of given two cells.*

For a more detailed discussion of SNN graph generation and its impact on subsequent clustering, see SNN Graph Determination in the supplementary paper.

### Binary Quadratic Model

The Binary Quadratic Model (BQM) formulation of the min-cut problem is presented below. As is illustrated in Table 1, we aim to find such values of linear and quadratic terms that will add the energy penalty when any two connected nodes are assigned to the separate clusters. This can be achieved by formulation of the objective function as presented in Equation (1), which will be minimised when neighboring cells belong to the same cluster.

Objective function: 


(1) \begin{align*}& min\sum_{(i,j)\epsilon E}(x_{i}+x_{j}-2x_{i}x_{j})\end{align*}

However, to prevent trivial solutions to the problem, such as assigning all nodes to a single cluster, we have to impose solution constraints, such as expecting sets to be of equal sizes. The constraint is defined in Equation (2), but we need to square it to ensure that the size violation energy penalty will always be positive. This results in Equation (3), which includes both quadratic and linear terms which can be mapped directly onto the Quantum Processing Unit (QPU). However, $ \left ( \sum _{i\epsilon V}x_{i} \right )^2 $ needs to be further rewritten and simplified: 


(2) \begin{align*} & \sum_{i\epsilon V}x_{i}=\frac{\left | V \right |}{2} \end{align*}

(3) \begin{align*} & \left ( \sum_{i\epsilon V}x_{i}-\frac{\left | V \right |}{2}\right )^2 \end{align*}

(4) \begin{align*} & \left(\sum_{i\epsilon V}x_{i}\right)^2+ \sum_{i\epsilon V}\sum_{j>i}2x_{i}x_{j}-\left | V \right |\cdot \sum_{i\epsilon V}x_{i}+\frac{\left | V \right |^2}{4} \end{align*}

(5) \begin{align*} \left ( \sum_{i\epsilon V}x_{i}\right)^2 & = x_{1}(x_{1}+x_{2}+x_{3}+...)+x_{2}(x_{1}+x_{2}+x_{3}+...) \nonumber\\ & \; \: +x_{3}(x_{1}+x_{2}+x_{3}+...)+... \nonumber\\ & = x^2_{1}+x^2_{2}+x^2_{3}+...+2x_{1}x_{2}+2x_{1}x_{3} \nonumber\\ & \; \; +2x_{1}x_{4}+2x_{2}x_{3}+2x_{2}x_{4}+... \nonumber\\ & = \sum_{i\epsilon V}x_{i} +\sum_{i\epsilon V}\sum_{j>i}2x_{i}x_{j} \end{align*}

**Table 1:** We want to add an energy penalty if two connected nodes are in different clusters. 0 and 1 in $x_i$ and $x_j$ columns represent distinct clusters.

$x_{i}$	$x_{j}$	Penalty
0	0	0
0	1	1
1	0	1
1	1	0

**Table 2:** We want to add an energy penalty if two connected nodes are in the same clusters. 0 and 1 in $x_i$ and $x_j$ columns represent distinct clusters.

$x_{i}$	$x_{j}$	Penalty
0	0	1
0	1	0
1	0	0
1	1	1

In Equation (5), we could reduce $x^2$ to $x$, as $0^2=0$ and $1^2=1$, so squaring doesn’t change the final energy result. Eventually, by combining the objective function with the constraint, we derive the final minimisation function, which will be embedded on the QA: 


(6) \begin{align*}& \sum_{(i,j)\epsilon E}(x_{i}+x_{j}-2x_{i}x_{j})+\gamma\left(\sum_{i\epsilon V}(1-\left | V \right |)x_{i}+\sum_{i\epsilon V}\sum_{j>i}2x_{i}x_{j}\right)\end{align*}

Gamma factor included in the constraint term regulates how much it will be respected. Therefore, keeping it high will bias solutions toward balanced partitions at the expense of sub-optimal graph cuts, while keeping it too low will result in excessively granular clustering. The exact value of the gamma factor is hard to predict accurately beforehand, as it depends on the graph connectivity and the character of the clustering that we want to find. However, we can easily tune it while keeping fixed problem embedding. Much more information on this topic can be found in the supplementary document.

### QA embedding

The defined BQM can be mapped to a QA, where the linear terms correspond to biases of single qubits, while the predefined Jaccoard similarity coefficients represent the quadratic terms.

**Listing 1:** Assignment of the linear and quadratic terms in the BQM matrix representation of the objective function.

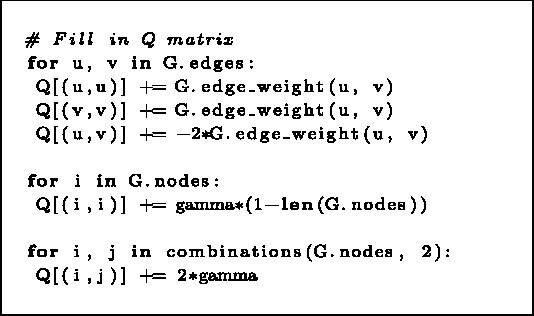

The next important point is setting up recursion-stopping conditions.The various approaches to this goal are discussed in detail in the supplementary document. In most cases, the best results were obtained when recursion was terminated based on the clustering confidence, which was determined by the energy variance of the most frequently sampled solutions. However, due to its high number of QPU calls (which access is limited on the monthly basic) for the results presented in this document, we have used CQM hybrid solver [19], which is also further discussed in the supplementary document.

**Figure 3:**
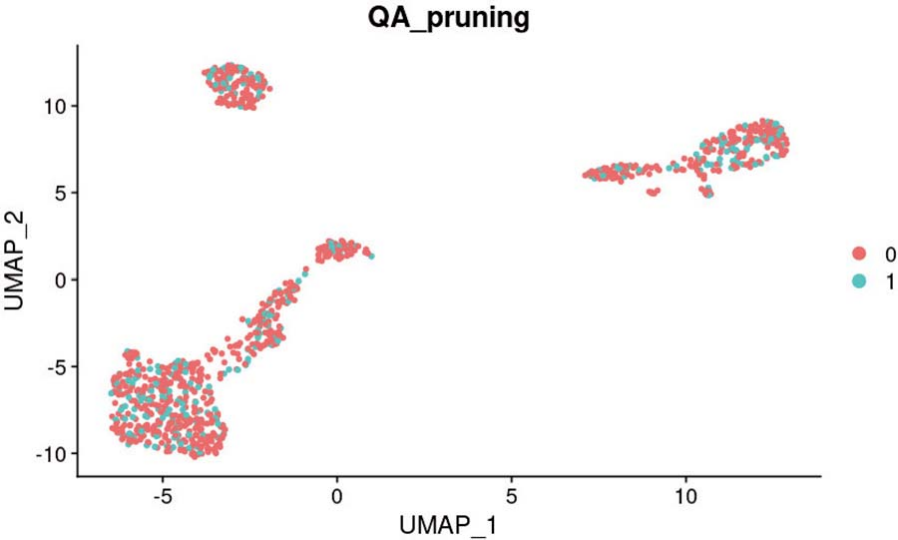
Minimum vertex cover problem evaluated on the QA The figure shows the solution of the minimum vertex cover problem (shown in the Uniform Manifold Approximation and Projection, UMAP, embedding), represented by a low-energy state sampled from a QA. The original graph has 1024 nodes. Blue points represent the selected sub-graph with 258 nodes.

**Figure 4:**
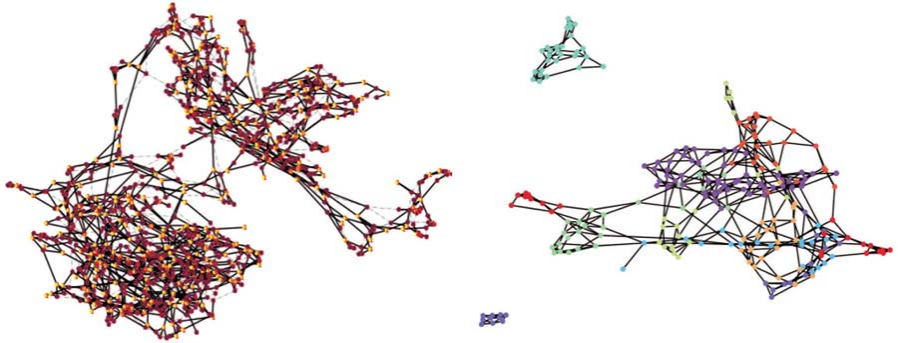
Quantum annealing-based clustering of the sub-sampled graph. The graph on the left represents a solution to the minimum vertex cover implemented on the QA. Yellow nodes correspond to the selected sub-graph. The graph on the right depicts partitioning of the SNN graph defined on the selected subset of nodes.

**Figure 5:**
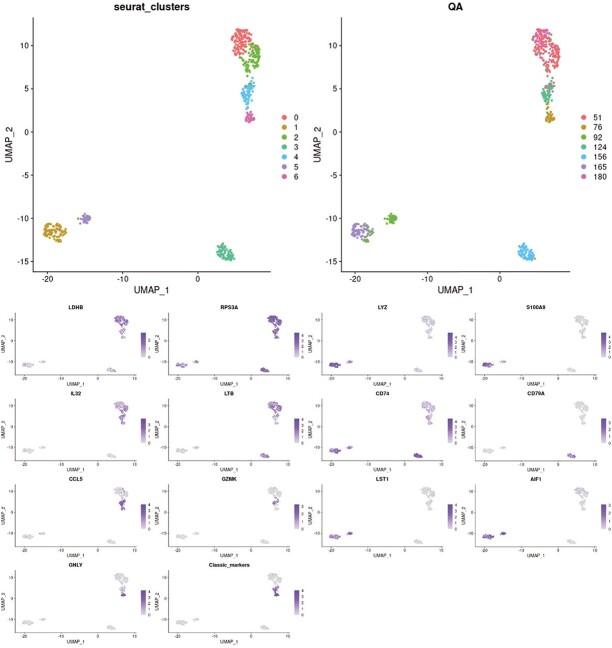
Data clustering results obtained using the state-of-the-art Seurat function and QA approach along with plots of the distribution of identified gene markers. We have used a subset of the pbmc3k data [20] consisting of 512 nodes. We see that the clusters found by the QA closely match the Seurat clusters. After examination of marker genes and reference cells types (which can be found in the appendix *CQM clusters verification*), one might claim that QA provides more accurate insight into data than the Seurat function, as it correctly hints about possible sub-types within identified clusters.

**Figure 6:**
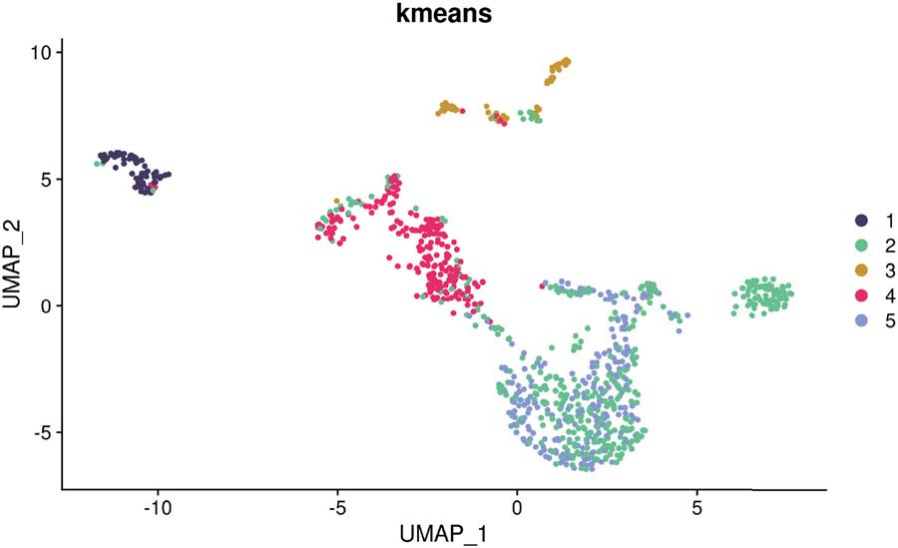
An example of K-means clustering visualized on UMAP projection of gene expressions of 1000 cells from the Human-Kidney dataset [16]. Compared with the projections illustrated in Figure 0, K-means can show poor performance in clustering which quantum annealing can potentially overcome.

### Graph sub-sampling

Importantly, as is presented in the supplementary document, due to limitations of currently available QPUs (quantum processing units), we are not able to use them directly for big scRNA-seq datasets. This can be overcome by evaluating only the most difficult (for classical algorithms) graph subsets, which often is an effective approach thanks to the general sparsity of the SNN graphs. Additionally, we can again employ quantum annealing to implement a minimum vertex cover problem, which reduces the number of samples while preserving as much of the information required for later clustering as possible. An example of this is presented in Figure 3, where blue nodes represent a solution to the minimum vertex cover problem sampled by the QA.

Minimum vertex cover is defined as a minimum-size subset of the nodes, V, such that each edge in E is incident to at least one vertex in this subset. It can be found by the minimisation of the model presented in Table 2.

Objective functions: 


(7) \begin{align*} & min\sum_{(i,j)\epsilon E}(1-x_{i}-x_{j}+x_{i}x_{j}) \end{align*}

(8) \begin{align*} & min\sum_{i\epsilon V}x_{i} \end{align*}

Final expression: 


(9) \begin{align*}& min\left ( \sum_{i\epsilon V}x_{i} + \gamma \sum_{(i,j)\epsilon E} (1-x_{i}-x_{j}+x_{i}x_{j})\right )\end{align*}

Equation (9) can be directly translated into the linear and quadratic terms of the BQM matrix. In this case, we do not impose any constraint on the subset sizes. Therefore, it significantly reduces the number of quadratic terms, which allows for the embedding of much bigger problems than for graph partitioning. Corresponding sizes of the selected sub-graphs can be adjusted while keeping fixed graph embedding, using the gamma factor. An example of the results obtained by the graph sub-sapling, and its subsequent partitioning, is presented in Figure 4. For a more detailed evaluation of this methodology and its verification against ground truth cells’ clusters, please refer to the supplementary document.

## CLUSTERS VALIDATION

There are many different metrics used to evaluate the accuracy of clustering. The main focus of these metrics is that the average intra-cluster distance and inter-cluster distance should be as small and large as possible, respectively. The Silhouette coefficient and Dunn index are two commonly used metrics that assess those features. However, in the case of scRNA-seq, where we often deal with clusters of different sizes and not symmetrical shapes, such classical metrics can be rather misleading. A good example is k-means clustering, which can perform relatively well in applied benchmarks, while its clusters are often very weakly correlated with actual cell types. Therefore, a more appropriate approach is to see if we can find uniquely expressed gene markers for the designated clusters. The appendix shows this in Figure 5, where after a closer examination of genes’ markers profiles among clusters, we can recognise that they correlate with the found partitioning.

With this in mind, we decided to verify the correctness of the clusters by directly comparing them with the curated cell types annotations. We used ground truth labels derived from experts’ domain knowledge instead of artificially generated labelled datasets. Since, in the former case, the cell type is assigned to each data point, based on a more accurate and precise study of the transcriptomic profile, while artificially generated data (e.g. based on RNA-seq generators or Gaussian mixture models) are subjected to their own biases (Gaussian mixture will have a non-zero probability of assigning any data to any cluster, and assumptions will underlie any model synthesizing RNA-seq data). For completeness, we included a list of standard clustering scores in the GitHub repository.

## CONCLUSION

QAs can efficiently and accurately sample low-energy solutions from complex energy landscapes, which correspond to alternative data clustering. Due to the computational overhead incurred by the generation of QPU embedding, this approach would not compete with classical counterparts for datasets of unambiguous partitioning. However, in the scRNA analysis, cluster assignment is often highly ambiguous as the cells’ identities evolve in pseudo-time and so the QA provides a unique advantage in generating a histogram of distinctive, high-quality partitioning together with associated energies. Such results are well suited for subsequent verification with biological knowledge and provide valuable insight into the heterogeneity of the analysed cell population. In contrast, to obtain similar results without the help of a QA, one would need to evaluate clustering repeatedly from different initial states and preferably with different algorithms to avoid imposing solution biases. Such solutions relying solely on classical algorithms come with a significant time cost compared with quantum annealing, which can generate hundreds of samples in milliseconds.

**Key Points**

- Quantum annealing offers a promising solution to efficiently sample potential scRNA-seq data clusters.
- This method reduces susceptibility to local minima, allowing for a more exhaustive exploration of the solution space compared with conventional clustering algorithms.
- While current limitations of quantum hardware prevent the direct analysis of real scRNA-seq datasets, problem sub-sampling and hybrid solvers can effectively overcome these limitations.

## CODE AVAILABILITY

A more detailed description of the methodology presented can be found in the supplementary document. Code, including data cleaning, pre-processing, different quantum annealing implementations and data visualisation, is published on GitHub: https://github.com/michal7kw/scRNA-seq-Clustering-QA.git.

## FUNDING

MN’s contribution to the work was funded by Engineering and Physical Sciences Research Council, UK: grant EP/S005463/1 ”Early detection of contact distress for enhanced performance monitoring and predictive inspection of machines.”

## Supplementary Material

QA_Clustering_supplement_bbad377
