# Supplementary material for: Quantum annealing-based clustering of single cell RNA-seq data
Source: Brief Bioinform. 2023 Oct 24;24(6):bbad377. doi: 10.1093/bib/bbad377 (PMC10597635; doi:10.1093/bib/bbad377)
Supplement: QA_Clustering_supplement_bbad377 [file qa_clustering_supplement_bbad377.pdf]

Journal Title Here, 2023, pp. 1–11

doi: DOI HERE
Advance Access Publication Date: Day Month Year
Paper

PAPER

Supplement: Quantum Annealing-based Clustering of Single Cell RNA-seq Data

Michal Kubacki[1,*] and Mahesan Niranjan[2,*]

[1, 2]Faculty of Engineering and Physical Sciences, University of Southampton
[*]Corresponding author. michal.kubacki11@gmail.com m.niranjan@southampton.ac.uk
FOR PUBLISHER ONLY Received on Date Month Year; revised on Date Month Year; accepted on Date Month Year

Abstract

This is a supplementary document to the paper "Quantum Annealing-based Clustering of Single Cell RNA-seq Data", which provides more details about different implementations and solution optimization strategies. For completeness and continuity of the message, some passages from the main text have been repeated.

General Problems in scRNA-seq Clustering

Data clustering is crucial and especially challenging step in the scRNA-seq analysis workflow(1). The main challenge stems from the fact that the analysed data are highly dimensional and often represent continuous cell differentiation pathways instead of separate clusters. In most cases, we also do not know the expected number of cell types in the sample, and the definition of when to assign a group to a distinct cell identity is not well defined. In the literature, one may encounter a number of different clustering methods, the most popular of which are listed below:

1. K-means clustering
2. Hierarchical clustering
3. Density-based clustering
4. Graph-based clustering
5. Mean shift clustering
6. Gaussian mixtures models

However, the correctness of the given clustering is highly objective as it depends on the semantics of the analysed data and the type of insight that we want to find. Therefore, there does not exist an ideal clustering method because different algorithms will emphasise different aspects of the analysed data. Thus, for example, one of the most popular clustering algorithms, k-means, results in compact and symmetric clusters, aiming to minimise within-cluster variances. Therefore, it is not well suited for the biological data where we expect not symmetric groups of the closely related cells as they progress in time. Also for the same reason, popular Gaussian mixture models, while more flexible in terms of cluster sizes and shapes, may still perform poorly for single-cell data(3). On the other hand, the clustering problem can be transformed into the problem of graph partitioning, where we do not assume any particular shape of the partitions. Thus, currently, many of the most popular clustering methodologies are implemented as the graph partitioning problems(1). Nevertheless, graph partitioning is a representative combinatorial optimization problem that is NP-hard. Therefore, exact solutions quickly become infeasible and we must resort to heuristic algorithms. However, due to the nature of scRNA-seq data, the loss function landscape corresponding to partitioning quality usually contains an abundance of locally optimal solutions representing potential cell classifications. Efficient optimization with such a complex landscape is inherently difficult and naturally leans toward probabilistic techniques such as those based on annealing.

In the literature, we can already find examples of simulated annealing applied to the graph partitioning problem(23). However, in this work we focus exclusively on the quantum annealer, as it has been shown to be a very promising alternative to simulated annealing capable of outperforming it in terms of both time and solution quality(24)(25)(26)(27).

Although gate model quantum computers of practical use are still a very distant prospect, quantum annealers (as offered by D-wave company), even nowadays, can be employed to solve real-life problems(7). They are designed to solve optimization problems and, in this respect, are superior to their gate-model counterparts(8)(9). In the literature, we can already find examples of successful applications of quantum annealing technology for life-science applications, such as peptides design(15)(16). Thanks to starting in the equal superposition and quantum tunnelling effect, we can much more extensively sample the loss energy function landscape, and so we are more likely to find the global minimum[1] or "difficult to access" local minima, which the classical simulated annealing would miss. Moreover, after the initial computational cost of problem embedding, with a minimal cost we can tune system parameters and sample a large number of low-energy states. Frequently occurring solutions will correspond to the

[1] due to inherent noise in the quantum systems, common approach to get exact minimum is to at the end of annealing refine found minima with the classical algorithms

© The Author 2023. Published by Oxford University Press. All rights reserved. For permissions, please e-mail: journals.permissions@oup.com

alternative partitioning, which, as will be discussed later, are invaluable in the case of scRNA-seq analysis.

Why Quantum Annealers?

One of the most promising applications for quantum annealers is to solve combinatorial optimisation problems. Therefore, the graph partitioning problem fits well to be solved using this new, emerging technology. As we mentioned in the previous paragraph, some of the popular methods used for data clustering made strong assumptions about the shapes of the clusters and their number. However, even when we decide to use, e.g. spectral clustering methods, which don't require such assumptions and can identify more unsymmetrical and subtle data points clusters. We are still faced with the problem that even theoretically optimal partitioning may lack biological interpretation, and some sub-optimal solutions may be much more faithfully related to the underlying biology(5)(4). Therefore, it is essential not to rely solely on methods that always result in a single answer (or closely related alternatives). But we instead seek a way to produce a distribution of possible solutions that biologists can later verify using already known facts such as cell cycles, genetic markers, and cell annotation(1). This, again, makes a perfect match with the quantum annelar based methods, as they can quickly sample a large number of low-energy states. Those states will correspond to the potential solutions that can be validated with the underlying biology. Additionally, given that the quantum annealer starts in the equal superposition at each run, found minimums may be very far from each other on the optimisation loss function landscape, which is not a case for many classical heuristic algorithms. This, in turn, will result in highly distinctive alternative assignments of clusters, possibly yielding better data insight.

Quantum Annealing Implementation

Graph partitioning can be solved through the recursive evaluation of the min-cut problem on the SNN graph. In this graph, nodes represent cells, and edges connect each node to their nearest neighbours with weights corresponding to the Jaccard similarity coefficient between a given pair of cells(6)(28). Although other similarity metrics may also be an appropriate choice, as shown in *Figure 3*, where we used k-Nearest Neighbor (kNN), calculated based on Euclidean distances. However, in general, we noticed that the SNN graph with weights defined as Jaccard indices performs better, so we decided to use it for our further investigation of the problem.

$$\textit{Jaccard Index between each pair of cells: } J(A,B) = \frac{|A \cap B|}{|A \cup B|}$$

where: A and B represent the binary differential expression values of given two cells

As is illustrated in the *Table 1*, we aim to find such values of linear and quadratic terms that will add the energy penalty when any two connected nodes are assigned to the separate clusters. This can be achieved by formulation of the objective function as presented in *Equation 1*, which will be minimised when neighbouring cells belong to the same cluster.

x_i	x_j	*penalty*
0	0	0
0	1	1
1	0	1
1	1	0

Table 1. We want to add an energy penalty if two connected nodes are in different clusters. 0 and 1 in x_i and x_j columns represent distinct clusters.

Objective function:

$$min \sum_{(i,j)\epsilon E} (x_i + x_j - 2x_ix_j) \qquad (1)$$

However, to prevent trivial solutions to the problem, such as assigning all nodes to a single cluster, we have to impose solution constraints, such as expecting sets to be of similar sizes. The constraint is defined in *Equation 2*, but we need to square it to ensure that the size violation energy penalty will always be positive. This results in *Equation 4*, which includes both quadratic and linear terms which can be mapped directly onto the Quantum Processing Unit (QPU). However, $(\sum_{i\epsilon V} x_i)^2$ needs to be further rewritten and simplified.

$$\sum_{i\epsilon V} x_i = \frac{|V|}{2} \qquad (2)$$

$$\left(\sum_{i\epsilon V} x_i - \frac{|V|}{2}\right)^2 \qquad (3)$$

$$(\sum_{i\epsilon V} x_i)^2 + \sum_{i\epsilon V}\sum_{j>i} 2x_ix_j - |V| \cdot \sum_{i\epsilon V} x_i + \frac{|V|^2}{4} \qquad (4)$$

$$\begin{aligned}\left(\sum_{i\epsilon V} x_i\right)^2 = \\ &= x_1(x_1 + x_2 + x_3 + \ldots) + x_2(x_1 + x_2 + x_3 + \ldots) \\ &+ x_3(x_1 + x_2 + x_3 + \ldots) + \ldots \\ &= x_1^2 + x_2^2 + x_3^2 + \ldots + 2x_1x_2 + 2x_1x_3 \\ &+ 2x_1x_4 + 2x_2x_3 + 2x_2x_4 + \ldots \\ &= \sum_{i\epsilon V} x_i + \sum_{i\epsilon V}\sum_{j>i} 2x_ix_j\end{aligned} \qquad (5)$$

In *Equation 5*, we could reduce x^2 to x, as $0^2 = 0$ and $1^2 = 1$, so squaring doesn't change the final energy result. Eventually, by combining the objective function with the constraint, we derive the final minimisation function, which will be embedded on the quantum annealer.

$$\sum_{(i,j)\epsilon E} (x_i + x_j - 2x_ix_j) + \gamma(\sum_{i\epsilon V}(1 - |V|)x_i + \sum_{i\epsilon V}\sum_{j>i} 2x_ix_j) \quad (6)$$

Gamma factor included in the constraint term regulates how much it will be respected. Therefore, keeping it high will bias solutions toward balanced partitions at the expense of sub-optimal graph cuts, while keeping it too low will result in excessively granular clustering.

Listing 1. Estimation of the gamma value.

```
edges_weights = G.size(weight="weight")
nodes_weights = len(G.edges)
ratio = edges_weights/nodes_weights
gamma = gamma_factor * ratio
```

The exact value of the gamma factor is hard to predict accurately beforehand, as it depends on the graph connectivity and the character of the clustering that we want to find. However, we can easily tune it while keeping problem embedding fixed.

Listing 2. Assignment of the linear and quadratic terms in the BQM matrix representation of the objective function.

```
# Fill in Q matrix
for u, v in G.edges:
 Q[(u,u)] += G.edge_weight(u, v)
 Q[(v,v)] += G.edge_weight(u, v)
 Q[(u,v)] += -2*G.edge_weight(u, v)

for i in G.nodes:
 Q[(i,i)] += gamma*(1-len(G.nodes))

for i, j in combinations(G.nodes, 2):
 Q[(i,j)] += 2*gamma
```

Figure 2 presents the histogram of solutions obtained for a graph with 128 nodes, each with 20 nearest neighbours (as presented in *Figure 1*). For the 5000 samples, we observe that the frequency of occurrence of the lowest energy state clearly surpasses all the others. The energy of this lowest energy state is equal to -16.39. As depicted in the *Figure 2*, the second optimal solution with the same energy exists, which corresponds to the opposite cluster assignment. However, it occurs much less often, which may be expected when considering the physics behind D-wave hardware where spin-down states are slightly more favourable than the spin-up states. Therefore, a larger cluster is more likely to end up in spin-down state.

The most time-consuming part of the Quantum Annealer solution is the determination of the problem's graph embedding. However, after this stage, we can keep the generated embedding fixed and solely select the particular nodes (representing the cells) of interest and, if required, modify the gamma factor and weights. This allows for the efficient recursion implementation and enables us to quickly adjust quadratic terms, e.g. if we know that particular cells are known to be far apart on the pseudo time trajectory. In our implementation, we defined edges' weights as the Jaccard similarity index, so the more similar cells are, the more biased they are toward a shared cluster. However, we may experiment with different distance metrics depending on analysed data or use the KNN graph's binary relations instead of continuous weights values.

As a terminating point of the recursion, we initially set the minimum viable size of a cluster. This straightforward method proved to work surprisingly well in the case of the pbmc3k dataset, mainly thanks to the fact that it contains distinct cell clusters. However, if the sequence data will include bigger groups of relatively similar cell types, this approach would not be optimal as the minimal cut will often result in sub-graphs of similar sizes. Thus, it will produce many recursive calls unnecessarily spending available QPU (Quantum Processing Unit) access time, and resulting in less informative over-granular clustering. Therefore, an alternative way is to examine, for each new partitioning, the energies of the

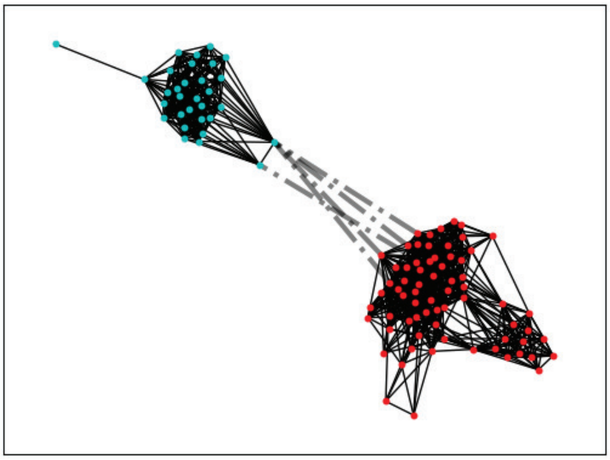

Figure 1. Graphical representation of the minimum cut solution. The figure illustrates a minimal cut solution obtained with Quantum Annealer, where dashed lines represent trimmed edges. The graph was defined as an SNN graph created for 128 cells from the pbmc3k dataset(29).

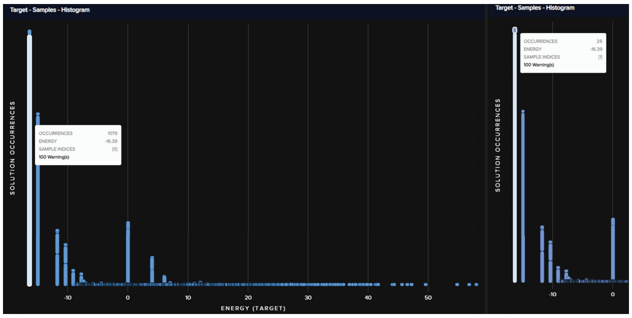

Figure 2. Histogram of the 5000 quantum annealer samples. Sampled quantum annealer states represent solutions to the minimum cut problem for a graph with 128 nodes (shown in *Figure 1*). The vertical axis corresponds to the number of occurrences of particular states, while the horizontal axis shows their energies. The small caption on the right side indicates an alternative solution of the same energy, which corresponds to the opposite cluster assignment.

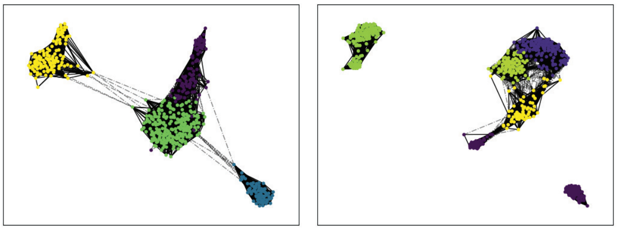

Figure 3. Results of quantum annealer graph clustering. The illustrated solutions were obtained for kNN and SNN (with weights defined as the Jaccard index) graphs, respectively, for the same data set.

top samples or their occurrence frequencies (which relate to configurations' energies). When particular partitioning is much more optimal than the other candidates (i.e. has distinctively smaller energy and is sampled much more often), we can accept the given solution and try to find another minimal cut among those new subgraphs. When there is no outstanding partitioning, we may assume that the considered cells are relatively homogeneous and belong to the same cell type. Thus we can think about such metric as the estimation of the solution confidence. However, due to the connectivity constraints, a given node often has to be represented by chains of qubits, which are strongly coupled, allowing them to operate as a single qubit. Therefore, if the energy differences imposed by the BQM matrix are comparable to or greater than the chain strengths, it can produce so-called chain breaks. In the results, we may get many identical solutions with slightly different energies (which would count as the distinct solutions) caused by the qubits flips within a single chain. Therefore obtained state counts will be misleading, wrongly indicating a homogenous cluster. It may seem that to solve this issue; we only need to set very high chain strengths. Unfortunately, this is not a solution as it will scale down problem imposed biases too much (all biases are scaled between -1 and 1), resulting in nodes adopting random states. Thus, when using "confidence" based stopping condition, we should remember to examine results carefully and choose chain strength appropriately. Fortunately, this value can be relatively easily estimated beforehand and later adjusted if needed, and D-wave libraries provide functions that do this automatically.
When it comes to the scalability of this approach, *Figure 4* depicts the actual embedding of the BQM problem on the quantum hardware, where quadratic terms are represented as the edges between nodes. This embedding was done for 128 nodes with 15 nearest neighbours each. Thus, even though the capabilities of QPUs are expected to grow substantially in the coming years. It is unlikely that we will be able to solve problems in the near future by directly emending BQM representations of problems for datasets with thousands of cells. However, they are many ways to bypass this limitation, using the methods described in the later sections.

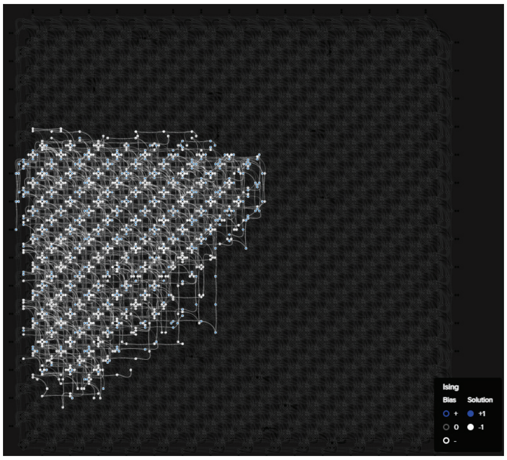

Figure 4. Visual representation of the Binary Quadratic Model embedded onto the quantum hardware. The figure presents the embedding of the clustering problem for an SNN graph with 128 nodes and their degrees trimmed to 15 on the D-wave Advantage QPU. Dots in the figure represent qubits (with spin states represented by white and blue colours), while white edges correspond to quadratic terms between nodes.

Graph Partitioning With Discrete Variables

It is also possible to formulate a minimal cut problem on quantum annealer for more than 2 clusters. This can be realised by the one-hot encoding of each partition, which on the hardware QPU embedding is represented as a very strong coupling between qubits corresponding to the single node, which enforces that only one particular state will be energetically feasible. This approach is presented and discussed in the later sections.

$$x = [x_{11} \ldots x_{N1} x_{12} \ldots x_{N2} \ldots x_{1k} \ldots x_{Nk}]^T \quad (7)$$

where: k-number of clusters, N-number of cells

SNN Graph Determination

When creating a dataset's SNN graph representation, there are multiple parameters to consider, which will influence, the size of the maximum graph which can be embedded, the computational cost of its determination and the characteristics of the found clusters. Those parameters include the used number of dimensions, distance metric, number of nearest neighbours, cutoff value and maximum allowed node degree. We will briefly discuss them below.

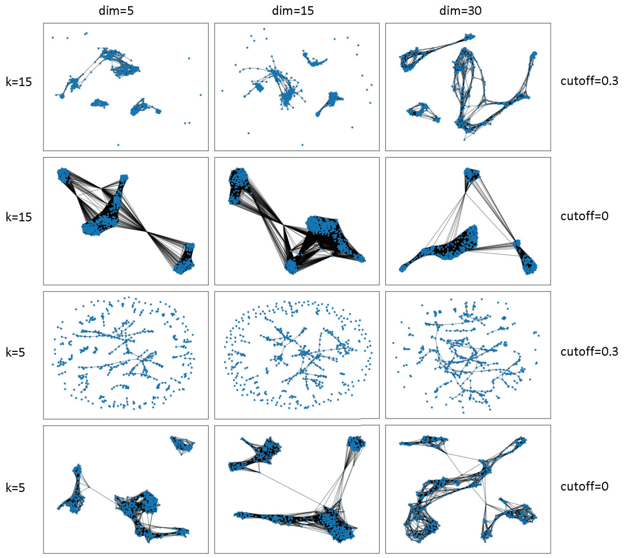

Figure 5. Influence of different parameters on the SNN graph structure. The figure presents how the choice of parameters used for graph generation influences their properties, where: *k*-number of nearest neighbours for which similarity index is calculated, *dim*-number of principal components used for similarity index determination, *cutoff*-cutoff value for similarly index. Graph were generated for the subset of 512 nodes from the pbmc3k dataset(29).

Cutoff value: Generation of the SNN graph with an appropriate high cutoff will result in the disconnected components representing cells with distinctive transcriptomic profiles. Disconnected communities can be found relatively efficiently using either BFS or DFS. Therefore, we may split the data using a classical computer and then sequentially send separate communities to the QPU, which will handle the classically computationally intensive graph partitioning

problem of closely related cells. However, too big cutoff value will result in many disconnected nodes losing the information about cells' correlations.

K-nearest neighbours: It specifies the number of nearest data points for which the quadratic term will be set. Setting it to a lower value will result in disconnected communities, which is advisable if one wants to focus separately on clusters of closely related cells. However, when this value is too small, the generated SNN graph will contain many small and disconnected sets, losing most information about cells' correlations. Graph generated with a bigger k value will be denser, therefore, more difficult to embed on the QPU with limited connectivity.

Maximum node degree: Even when we define SNN with a relatively small number of the nearest neighbours, the nodes lying within tight clusters will be among the nearest neighbours of many other cells and, therefore, may have a very high degree. This initially may seem to be beneficial for the graph partitioning problem as it increases the energetical benefits of clusters. However, high-degree nodes are more resource expensive and difficult to embed on QPU (this is not so significant for the direct embedding of the Constrained Binary Model, as will be explained in the section: *Unconstrained Binary Model*). Furthermore, as shown in *Figure 6*, untrimmed connectivity can be undesirable in most cases, as it overestimates the value of nodes in large clusters, which has a negative impact on more subtle cluster recognition.

Dimensions: It defines how many principal components are used to evaluate the similarity between given data points. Setting it to a higher value enables identifying the more subtle correlations between the cells. Therefore, as shown in *Figure 5*, this results in much more far-reaching graph connectivity, enabling a better assessment of cluster correlations.

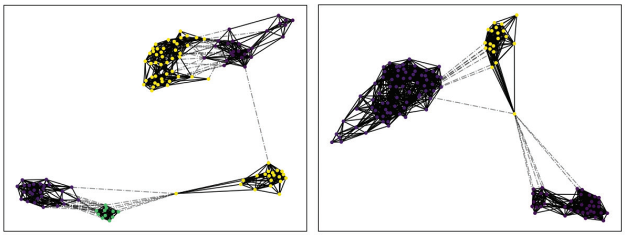

Figure 6. Influence of graph connectivity density on the partitioning results. The figure compares clustering results obtained for graphs with trimmed and not-trimmed nodes' degrees, respectively.

When we further consider the characteristics of scRNA-seq data. We may suspect that there are many points in between clusters that would correspond to the transition states. In addition, taking into account the evolution of the genes expression landscapes, prolonged distributions of the cells of the close Jaccard similarity are expected, which eventually will result in stable and bigger populations. So to facilitate clusters determination, we may want to amplify connection weights between the nodes, which are both among their corresponding k-nearest neighbours. Such a procedure is very efficient, as it consists of easily parallelisable operations on the graph's adjacent matrix[2].

Clusters Validation

There are many different metrics used to evaluate the accuracy of clustering. The main focus of these metrics is that the average intra-cluster distance and inter-cluster distance should be as small and large as possible, respectively. These objectives can be further divided into three categories: Compactness - measures how close are the objects within the same cluster, Separation - measures how well-separated a cluster is from other clusters, Connectivity - corresponds to what extent items are placed in the same cluster as their nearest neighbours in the data space. The Silhouette coefficient and Dunn index are two commonly used metrics that assess those features. However, in the case of scRNA-seq, where we often deal with clusters of different sizes and not symmetrical shapes, such classical metrics can be quite misleading. A good example is k-means clustering, which may achieve relatively good scores across applied benchmarks. However, those clusters often weakly correlate to the actual cell types. For completeness, we included a list of standard clustering scores in GitHub repository and visual comparison with other clustering methods in the appendix: *Figure 20*. A more appropriate approach would be to check whether we can find uniquely expressed gene makers for the determined clusters. This is illustrated in *Figure 16*, where, after a closer examination of genes' markers profiles among clusters, we can recognise that they correlate with the found partitioning. In the rest of our work, we used annotated datasets, in which cell type is assigned to each data point, based on a more thorough and accurate transcriptomic profile examination. Therefore, we verified the found clusters' correctness in the following sections by directly comparing them to the curated cell types annotations as presented in *Figure 17*.

Unconstrained Binary Model

After a close examination of the currently used BQM model, it is apparent that most of the embedding costs are introduced by the quadratic terms of the cluster balancing constraint. This problem is illustrative when inspecting the actual embedding on the QPU. We see there that each node is represented by the long chains of qubits, often spanning throughout the whole chip. This is because the imposed constraint puts bias between each pair of nodes. Therefore, even with the relatively sparse graphs, in which the nodes' degrees are trimmed to only 5, the current D-wave Advantage system offering 5000 qubits, with 15 degrees each, is only able to accommodate graphs with approximately 128 nodes. This value is not strictly defined as the embedding algorithm is heuristics, so it may vary slightly between the runs.

A potential solution to this problem is to modify the balancing constraint, as in the end, we don't aim for perfectly balanced clusters and currently implement code accounts for possible clusters of very different sizes by additional gamma coefficient, which reduces the penalty for the clusters sizes equality violation. Here, we will briefly describe two potential approaches, namely unconstrained model and hybrid solver. In the first instance, we completely remove the quadratic

[2] Implemented can be checked in the published GitHub repository

term and only impose a tiny linear bias to all the qubits, which will impose an energy penalty for each qubit in the "1" state, therefore preventing the system from ending entirely in a single cluster. Although this approach is very hardware efficient, it requires precise adjustment of chain strengths and the ratio of quadratic and linear biases. Those values can be approximated beforehand, ensuring that overall linear terms will be of a similar order of magnitude as quadratic terms, but subsequent empirical tuning is still required. Fortunately, this can be performed while keeping the graph embedding intact so that it might be accomplished in a relatively efficient manner. Additionally, QPU returns a histogram of obtained energy values after each run, which provides useful guidance for parameters optimisation. Defining gamma factor as shown in *Listing 3* with the adjustable *gamma_factor* term proved to work well in the case of the analysed dataset.

Listing 3. Assignment of the linear and quadratic terms in the BQM matrix without accounting directly for the balancing constraint.

```
# Fill in Q matrix
for u, v in G.edges:
  Q[(u,u)] += k*G.edge(u, v)["weight"]
  Q[(v,v)] += k*G.edge(u, v)["weight"]
  Q[(u,v)] += k*-2*G.edge(u, v)["weight"]

for i in G.nodes:
  Q[(i,i)] += gamma
```

Listing 4. Code for initial estimation of the gamma value.

```
nodes_len = len(G.nodes)
degrees = list(nodes_degrees(G))
d_mean = np.mean(degrees)

weights = list(all_edges_weights(G))
w_sum = G.size(weight="weight")
w_mean = np.mean(weights)

chain_strength = w_mean*d_mean*2
gamma = (w_sum/nodes_len)*gamma_factor
```

We need to be carefully with adjusting the gamma factor, as in this case, for the relatively sparse graphs, the number of linear biases will be much greater than the number of quadratic terms. Therefore, we need to use a very small coefficient for linear terms compared to quadratic ones. Although, such a big span of biasing values due to the current inaccuracies and limitations of quantum hardware is likely to introduce a lot of noise to our clustering results. An alternative way would be to increase graph density significantly, which would allow to partially reduce differences between the sizes of linear and quadratic terms. However, this will decrease the size of the maximal realisable size of the embedding. Some of the results obtained using discussed here approach, showing the significant influence of noise and means to suppress it are presented in *Figure 18*, while *Figure 7* shows depictions of the actual problem embedding onto the quantum annealing hardware.

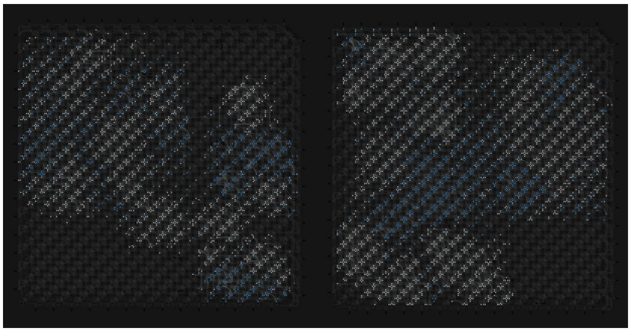

Figure 7. Hardware embedding of the binary optimisation problem. The figure illustrates an unconstrained optimisation problem embedding defined for a graph with 512 nodes. The graph on the left was constructed with the 5, while the graph on the right with the 15 nearest neighbours. Thanks to the 15-degree connectivity of each qubit (in the D-wave Advantage processor), the three-fold increase in connectivity only slightly increased embedding size.

Hybrid solvers

The methods described above allow fine control of the characteristics of the final solution, but require manual tuning of the parameters, and due to the recursive implementation they generate a large number of separate QPU calls, the number of which is still quite limited at this early stage of quantum computing development. Moreover, in most situations, the dataset of interest will be too large to be directly embedded on the QPU using the method described above. To overcome this size limitation, D-wave provides proprietary hybrid solvers that make use of both classical and quantum resources to solve problems, exploiting the complementary strengths that each provides(37). Developed frameworks based on limited information about the full-sized problem can generate useful suggestions about promising regions of the search space to explore. It does so by using the decomposer to split the original problem into sub-problems by selecting only a part of the variables. One of the classical decomposition approaches, so-called energy-based selection, selects first n variables that have the highest impact on the objective function. Then it combines sampled solution of the multiple sub-problems to generate the final answer. For more information on available hybrid solutions and their advantages over purely classical methods, please refer to the following resources(36)(35). At the time of writing, there are three different hybrid solvers supported by the D-wave platform(37):

Hybrid BQM solver: Binary quadratic models (BQM)(32) typically represent problems of decisions that could either be true or false. These solvers accept arbitrarily structured, unconstrained problems formulated as BQMs, with any constraints typically represented through penalty models(37). The solution presented in *Figure 16* was obtained by recursive evaluation of Binary quadratic models on the Hybrid BQM solver.

Hybrid DQM solver: Discrete quadratic models (DQM)(34) typically represent problems with several distinct options. These solvers accept arbitrarily structured, unconstrained problems formulated as DQMs, with any constraints typically represented through penalty models(37). At the time of writing, this model supports up to 3000 variables, and as is presented in

Figure 8, it could correctly identify cell clusters. However, as in this case, we are not able to choose the gamma factor, and the DQM hybrid solver enables only the equality constraints; it will be biased toward clusters of necessarily equal sizes. Therefore, for most cases where clusters are expected to be of significantly different sizes CQM model supporting inequality constraints will be more advisable.

Hybrid CQM solver: Constrained quadratic models (CQM)(33) typically represent problems that might include real, integer or binary variables and one or more constraints. These solvers accept arbitrarily structured problems formulated as CQMs, with any constraints represented natively(37). This Hybrid solver improves on the previous approach as it enables inequality constraint and at the time of writing it supports up to 5000 variables. Thus, e.g. could use to run clustering for 512 nodes with 9 clusters (4608 variables), the results of which are shown in *Figure 9*. Problem formulation, which is solved by the CQM hybrid solver, is presented in *Listing 5*.

Listing 5. CQM implementation of graph partitioning problem.

```
print("Adding variables")
  v = [[dimod.Binary(f'v_{i},{k}')
    for k in clusters]
      for i in nodes]

print("Adding one-hot constraints...")
for i in nodes:
  cqm.add_discrete([f'v_{i},{k}'
    for k in clusters],
     label=f"one-hot-node-{i}")

print("Adding objective")
min_edges = []
for i,j in edges:
 for p in clusters:
  min_edges.append(
   v[int(i)][p]+v[int(j)][p]
   - 2*G.get_edge_data(i, j)["weight"]
   *v[int(i)][p]*v[int(j)][p])
cqm.set_objective(sum(min_edges))

print("Adding partition size constraint")
 for j in clusters:
  cqm.add_constraint(
   sum(v[int(i)][j] for i in nodes) >= 20,
    label=f'cluster_size{j}')
```

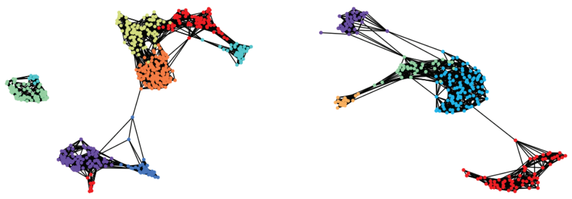

Figure 8. DQM solutions to graph partitioning problem. The figure shows graph partitioning solutions obtained using DQM hybrid solver for datasets of 512 and 256 cells, respectively.

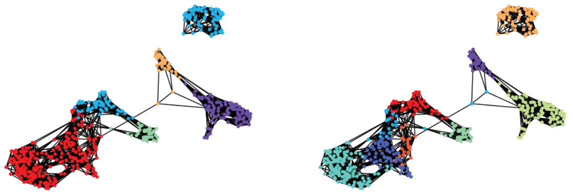

Figure 9. CQM solutions for the graph partitioning problem. The figure shows the graph partitioning solutions obtained with the CQM hybrid solver for a dataset of 512 cells. The hybrid solver was configured for 7 and 9 clusters, respectively. Evaluation of validity of the found graph partitions and comparison to the Seurat clustering algorithm are presented in *Figure 17*.

Graph Subsampling

CQM or DQM are currently bounded by 5000 and 3000 variables, respectively, which is still too little for many scRNA-seq datasets. Evaluating problems recursively using a hybrid solver fixes these limitations and is expected to achieve high accuracy thanks to the sparsity of the generated SNN graphs. However, access time to the hybrid solver can be relatively limited at present, and its multiple calls at each recursion step quickly consume available QPU access time. Therefore, to reduce the number of samples, we implemented a minimum vertex cover problem on the QA. This significantly reduces the number of nodes by sampling out cells which are close together and so less informative to the overall partitioning while preserving the most significant ones. An example of this is presented in *Figure 10*, where blue nodes represent a solution to the minimum vertex cover problem sampled by the quantum annealer. Discarded points will be later incorporated into the found clusters by assigning them to the closest group.

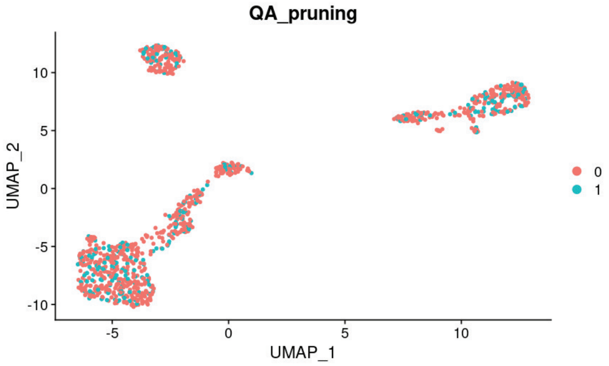

Figure 10. Minimum vertex cover problem evaluated on the quantum annealer. The figure shows the solution of the minimum vertex cover problem (shown in the UMAP embedding), represented by a low-energy state sampled from a quantum annealer. The original graph has 1024 nodes. Blue points represent the selected sub-graph with 258 nodes.

Minimum vertex cover is defined as a minimum-size subset of the nodes, V, such that each edge in E is incident to at least one vertex in this subset. It can be found but the minimisation of a model presented in *Table 2*.

x_i	x_j	*penalty*
0	0	1
0	1	0
1	0	0
1	1	1

Table 2. We want to add an energy penalty if two connected nodes are in the same clusters. 0 and 1 in x_i and x_j columns represent distinct clusters.

Objective functions:

$$min \sum_{(i,j)\epsilon E} (1 - x_i - x_j + x_i x_j) \quad (8)$$

$$min \sum_{i\epsilon V} x_i \quad (9)$$

Final expression:

$$min \left(\sum_{i\epsilon V} x_i + \gamma \sum_{(i,j)\epsilon E} (1 - x_i - x_j + x_i x_j) \right) \quad (10)$$

Equation 10 can be directly translated into the linear and quadratic terms of the BQM matrix. In this case, we do not impose any constraint on the subset sizes. Therefore, it significantly reduces the number of quadratic terms, which allows for the embedding of much bigger problems than for graph partitioning. Corresponding sizes of the selected subgraphs can be adjusted while keeping fixed graph embedding, using the gamma factor. An example of the results obtained by the graph sub-sapling, and its subsequent partitioning, is presented in *Figure 11*.

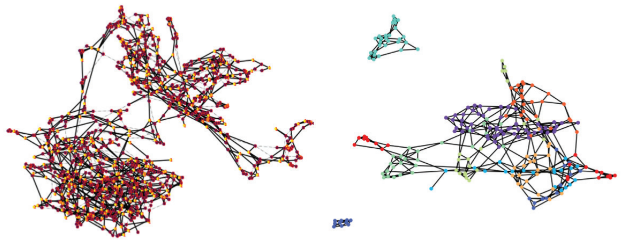

Figure 11. Quantum annealing-based clustering of the sub-sampled graph. The graph on the left represents a solution to the minimum vertex cover implemented on the quantum annealer. Yellow nodes correspond to the selected subgraph. The graph on the right depicts partitioning of the SNN graph deffined on the selected subset of nodes.

Results

This section describes obtained results for two different example detests. Which are the sub-parts of Kidney Cell Atlas(2).

Experiment 1

Quantum annealing based clustering yields the most significant promise of advantage for the relatively homogeneous clusters. As in those cases, classical algorithms are prone to settle at local minimums and miss more subtle cells partitioning. Therefore, here we used subset of the Kidney cells data set(2), which consists of groups of closely related cell types, and so is much harder to examine correctly using classical clustering algorithms. Selected subset includes five different cell types: Ascending vasa recta endothelium, Descending vasa recta endothelium, Pertibular capillary endothelium 1, Pertibular capillary endothelium 2, and Myofibroblast.

After an inspection of the results presented in *Figure 12* we see that the Seurat clustering function and the most frequently occurring state of the quantum annealer found different sets of clusters, which both correctly correlate with the cell identities. However, non of them indicates the whole heterogeneity of the population. Thus, for example, the Seurat clustering function failed to distinguish between peritubular capillary endothelium and ascending vasa recta endothelium cells. At the same time, the lowest energy state found by the annealer did not indicate heterogeneity in the population of peritubular capillary endothelium cells. In the case of the classical algorithm, to get a further insight into data, we need to rerun the whole clustering from the beginning, e.g. with slightly changed clustering parameters or distance metrics. However, in many cases, this will result in very similar, and so not much more informative, data partitioning. The situation is much different for the QA, where we can just look at the other low energy states, which have been sampled most frequently. As the *Figure 12* shows, the alternative solutions found by the quantum annealer correctly hint about additional sub-populations. Moreover, as discussed earlier, there may be not something like ideal clustering, so we shouldn't trust the single clustering result. Instead, biologists would like to see a couple of clustering variants that could be reviewed more closely later and that would help provide more insight into the data. In addition, to further explore data, we may emphasise a different aspect of it by tweaking annealing coefficients for each sampling run (this can be done very efficiently, as it doesn't require a generation of new embedding). *Figure 13* shows the found sets of clusters corresponding to successive low-energy states sampled by the quantum annealer, with significant heterogeneity of the found partitions apparent.

Experiment 2

To further validate the advantage of QA for scRNA data, we selected another cells subset consisting of 5 different cell types, among which were two closely related pairs. Selected cells are presented in the *Figure 14*.
As *Figure 14* presents Seurat clustering method failed to distinguish distinct cell identities of the closely related pairs. To evaluate this problem on a quantum annealer, we used a hybrid CQM solver with a one-hot encoding of five different clusters, meaning that the maximum number of cells we could represent on a QPU was 1000. However, the selected data consisted of 2901 cells, so we had to use the data sub-sampling method discussed in the section on Graph Sub-sampling. The max vertex cover problem, evaluated on the quantum annealer, picked the subset of 942 nodes, which clustering could then be solved on the QPU.

However, as depicted in *Figure 14*, QA the lowest energy solution similarly to Seurat clustering function also fails to distinguish between two sub-types of neutral killer cells. Fortunately, the same as in the last example, we can simply examine other low energy state (shown in *Figure 15*), which clearly indicates transcriptomic profile differences within the population of those two cell types. Visualisations of graph partitions corresponding to those other energy states are presented in *Figure 19*.

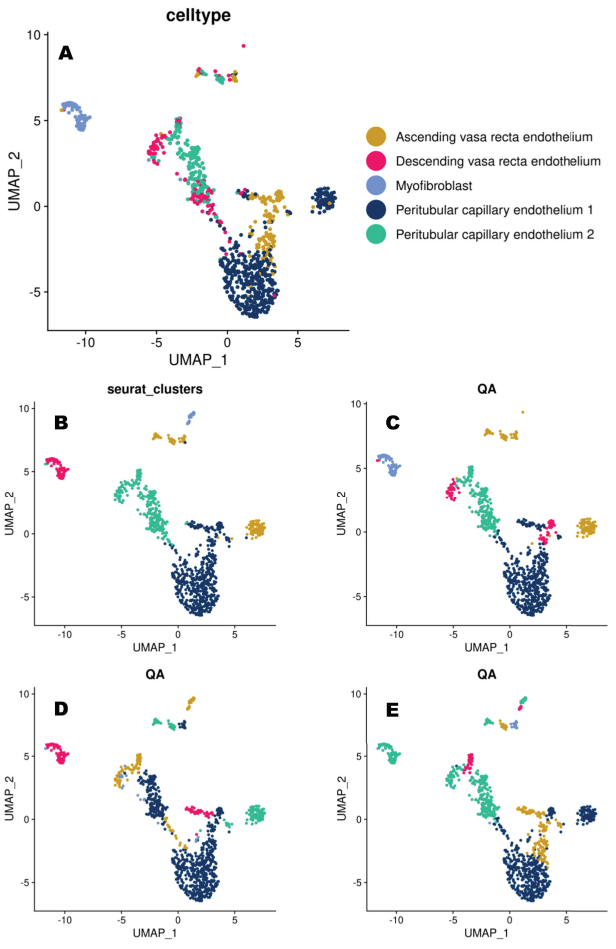

Figure 12. **The advantage of quantum annealing in the study of population heterogeneity.** Sub-figure *A* shows the original cells' types annotations. Then in order, Seurat(30) clusters and three of the low energy states found by the quantum annealer are presented. Analysis of the spectrum of potential solutions sampled by the quantum annealer provides better insight into cells population than a single classical solution. Clusters' colors in the plots serve only visualisation and do not directly relate to a particular cell type.

Run-time

Due to still the early years of quantum computing technology direct access to the quantum processing unit is relatively limited and so the actual time spent on quantum annealing (using the CQM solver) is 16 ms. Therefore, it is reasonable to imagine a significant extension of QA sampling time, which might yield much more optimal solutions, and help to suppress the effect of intrinsic system noise. However, the total run-time is longer and accounts for 5 seconds, and it's mostly due to web API delays and the time required for an initial problem embedding.

The recursive method described here, due to the generation of many separate problem submissions, increases the execution time (depending on the clustering granularity sought), although it still works within a very practical time-frame of at most a few minutes in total.

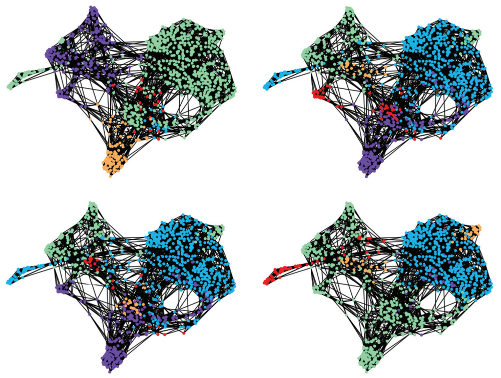

Figure 13. Examples of the first four lowest energy states returned by the quantum annealer on 1000 nodes. These are low-energy states of the clustering cost function showing considerable heterogeneity, suggesting multiple useful (low-energy) solutions can be obtained, which will not be the case for classical algorithms like K-means without considerable computing starting from a large number of initial conditions. The colours serve only visualisation and do not directly relate to a particular cell type.

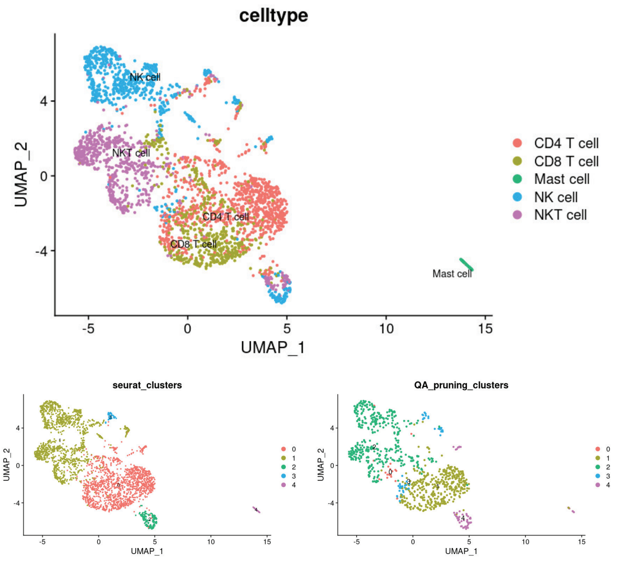

Figure 14. Comparison of Seurat's solution and QA of the lowest energy state with ground truth cell types. The figures at the bottom show results of the Seurat clustering function run on the original dataset (2901 nodes) and the most frequently sampled QA solution for the pruned graph (942 nodes). Clusters' colors in the plots serve only visualisation and do not directly relate to a particular cell type.

Conclusion

Proposed here, data clustering implementation on quantum annealer provides an excellent alternative to classical clustering methods, as it can very efficiently sample low energy solutions corresponding to the alternative clusters assignments. This approach would not compete with classical counterparts for datasets with clearly separated cell types due to the computational cost of generating QPU embedding and unambiguity of the optimal clusters assignment. However, it may provide much better data insight in a shorter time than classical algorithms for datasets of closely related cells.

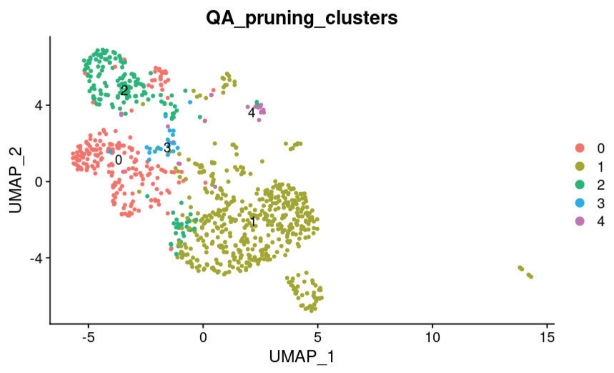

Figure 15. Clustering defined by the second lowest energy state found with QA. The figure shows data clustering corresponding to the second most frequently occurring configuration found by the quantum annealer. And comparing to the first lowest energy solution, it indicates an additional cell sub-population which correctly relates to the ground truth values. Clusters' colors in the plots serve only visualisation and do not directly relate to a particular cell type.

Code Availability

Additional, information and code including: data cleaning, data pre-processing, different quantum annealing implementations, and data visualisation; are published on GitHub. Importantly to reproduce the pipeline one will need to acquire Dwave API Token, which is available for free after login into D-wave Leap portal.

Competing interests

No competing interest is declared.

References

1. Kiselev, V., Andrews, T. and Hemberg, M., 2019. Challenges in unsupervised clustering of single-cell RNA-seq data. Nature Reviews Genetics, 20(5), pp.273-282.
2. Stewart, B. and Ferdinand, J., 2019. Spatiotemporal immune zonation of the human kidney. Science, 365(6460), pp.1461-1466.
3. Liu, Z. (2021) "Clustering single-cell RNA-seq data with regularized Gaussian Graphical Model," Genes, 12(2), p. 311.
4. Schmidt, F. and Ranjan, B. (2021) "Robust clustering and interpretation of scrna-seq data using reference component analysis."
5. Wu, Z. and Wu, H. (2020) "Accounting for cell type hierarchy in evaluating single cell RNA-seq clustering," Genome Biology, 21(1).
6. Levine, J.H. and Simonds, E.F. (2015) "Data-driven phenotypic dissection of AML reveals progenitor-like cells that correlate with prognosis," Cell, 162(1), pp. 184–197.
7. D-Wave Whitepaper Series: Practical Quantum Computing, 2020-10-07
8. Crosson, E. and Harrow, A., 2016. Simulated Quantum Annealing Can Be Exponentially Faster Than Classical Simulated Annealing. 2016 IEEE 57th Annual Symposium on Foundations of Computer Science (FOCS).
9. Guerreschi, G. and Matsuura, A., 2019. QAOA for Max-Cut requires hundreds of qubits for quantum speed-up. Scientific Reports, 9(1).
10. Chen, H., Guo, J., Mishra, S., Robson, P., Niranjan, M. and Zheng, J., 2014. Single-cell transcriptional analysis to uncover regulatory circuits driving cell fate decisions in early mouse development. Bioinformatics, 31(7), pp.1060-1066.
11. Farran, B., Ramanan, A. and Niranjan, M., 2009. Sequential Hierarchical Pattern Clustering. Pattern Recognition in Bioinformatics, pp.79-88.
12. Frey, B. and Dueck, D., 2007. Clustering by Passing Messages Between Data Points. Science, 315(5814), pp.972-976.
13. Arthur, D. and Date, P., 2021. Balanced k-means clustering on an adiabatic quantum computer. Quantum Information Processing, 20(9).
14. Ushijima-Mwesigwa, H., Negre, C. and Mniszewski, S., 2017. Graph Partitioning using Quantum Annealing on the D-Wave System.
15. Mulligan, V., Melo, H., Merritt, H., Slocum, S., Weitzner, B., Watkins, A., Renfrew, P., Pelissier, C., Arora, P. and Bonneau, R., 2019. Designing Peptides on a Quantum Computer.
16. Mato, K., Mengoni, R., Ottaviani, D. and Palermo, G., 2022. Quantum molecular unfolding. Quantum Science and Technology, 7(3), p.035020.
17. Bodlaender, H.L., Jansen, K. "On the complexity of the maximum cut problem." Enjalbert, P., Mayr, E.W., Wagner, K.W. (eds) STACS 94. STACS 1994. Lecture Notes in Computer Science, vol 775. Springer, Berlin, Heidelberg.
18. G. G. Guerreschi, A. Y. Matsuura. "QAOA for Max-Cut requires hundreds of qubits for quantum speed-up.
19. Catherine F. Higham, Desmond J. Higham, and Francesco Tudisco. "Testing a QUBO Formulation of Core-periphery Partitioning on a Quantum Annealer."
20. Tim Jaschek, Marko Bucyk, and Jaspreet S. Oberoi. "A Quantum Annealing-Based Approach to Extreme Clustering."
21. Hayato Ushijima-Mwesigwa, Christian F. A. Negre, and Susan M. Mniszewski. "Graph Partitioning using Quantum Annealing on the D-Wave System."
22. Wang, B., Hu, F., Yao, H. et al. "Prime factorization algorithm based on parameter optimization of Ising model" Sci Rep 10, 7106 (2020).
23. Johnson, D.S., Aragon, C.R., McGeoch, L.A. and Schevon, C. (1989). Optimization by Simulated Annealing: An Experimental Evaluation; Part I, Graph Partitioning. Operations Research, 37(6), pp.865–892.
24. Crosson, E. and Harrow, A.W. (2016). Simulated Quantum Annealing Can Be Exponentially Faster than Classical Simulated Annealing. 2016 IEEE 57th Annual Symposium on Foundations of Computer Science (FOCS), pp.714–723.
25. Boixo, S., Rønnow, T.F. and et al. (2014). Quantum annealing with more than one hundred qubits. Nature Physics, 10(3), pp.218–224.
26. Koshka, Y. and Novotny, M.A. (2019). Comparison of D-Wave Quantum Annealing and Classical Simulated Annealing for Local Minima Determination.
27. Nishimori, H. (2015). Comparison of quantum annealing and simulated annealing. The European Physical Journal Special Topics, 224(1), pp.15–16.
28. Package 'Seurat
29. https://www.10xgenomics.com/resources/datasets
30. https://cran.r-project.org/web/packages/Seurat/Seurat.pdf
31. https://www.dwavesys.com

32. https://www.dwavesys.com/media/soxph512/hybrid-solvers-for-quadratic-optimization.pdf
33. https://www.dwavesys.com/media/rldh2ghw/14-1055a-a_hybrid_solver_for_constrained_quadratic_models.pdf
34. https://www.dwavesys.com/media/ssidd1x3/14-1050a-a_hybrid_solver_for_discrete_quadratic_models.pdf
35. https://docs.ocean.dwavesys.com/_/downloads/hybrid/en/latest/pdf/
36. https://docs.ocean.dwavesys.com/en/stable/overview/hybrid.html
37. https://docs.dwavesys.com/docs/latest/doc_leap_hybrid.html

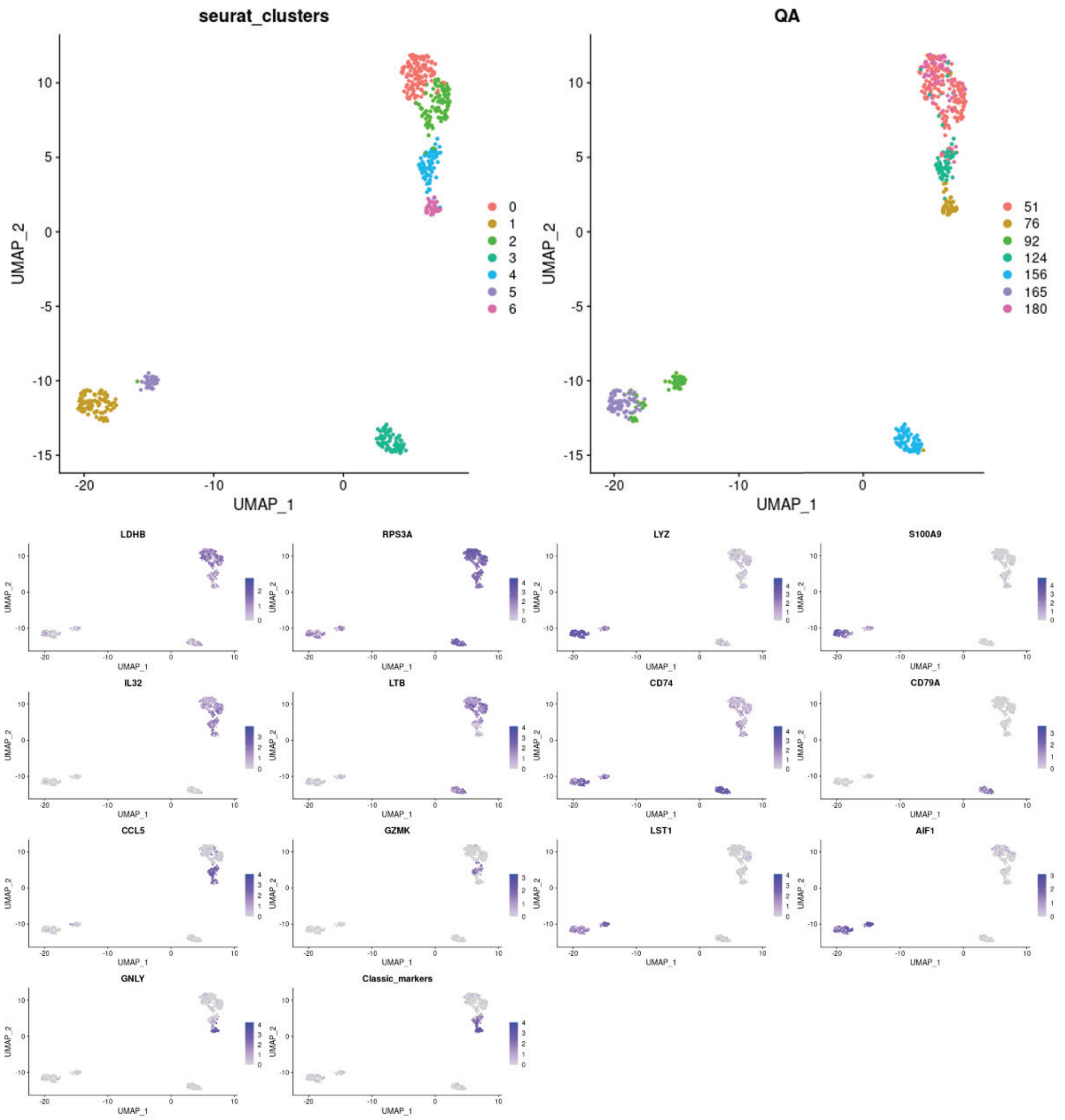

Figure 16. Data clustering results obtained using the state-of-the-art Seurat function and QA approach along with plots of the distribution of identified gene markers. We have used a subset of the pbmc3k data(29) consisting of 512 nodes. We see that the clusters found by the QA closely match the Seurat clusters. After examination of marker genes and reference cells types (which can be found in the appendix *CQM clusters verification*), one might claim that QA provides more accurate insight into data than the Seurat function, as it correctly hints about possible sub-types within identified clusters.

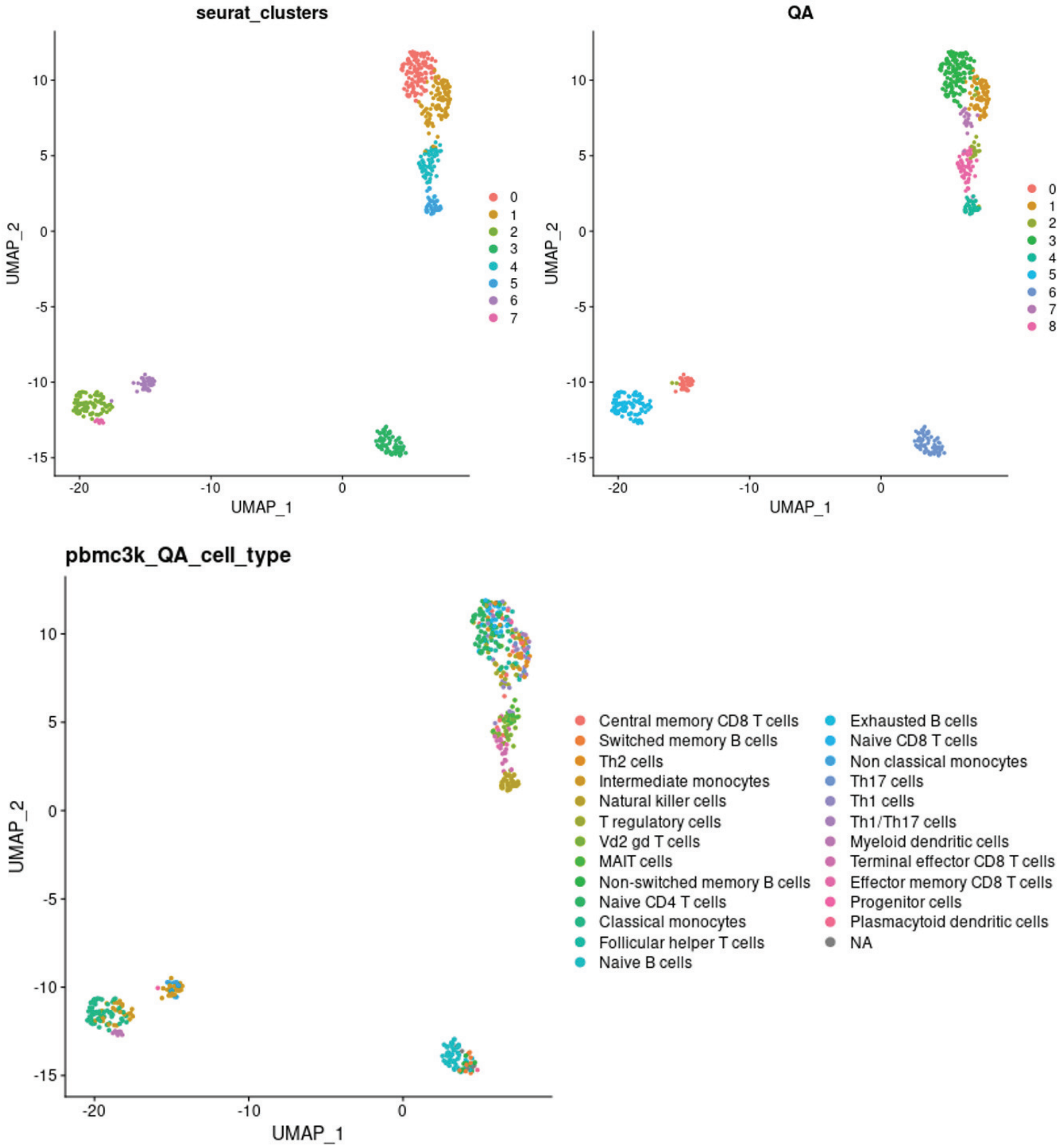

Figure 17. Comparison between CQM and Seurat clustering. When comparing both of the solutions with the cell annotations, we can recognize that additional clusters found using QA correspond to the Th1/Th17 and Vd2 cells. The solution found by the Seurat clustering shown in this figure is slightly different from the one shown in *Figure 16*, because we changed its "resolution" parameter. Which caused it to find a more granular clustering that was more suitable for comparison with curated cell type labels.

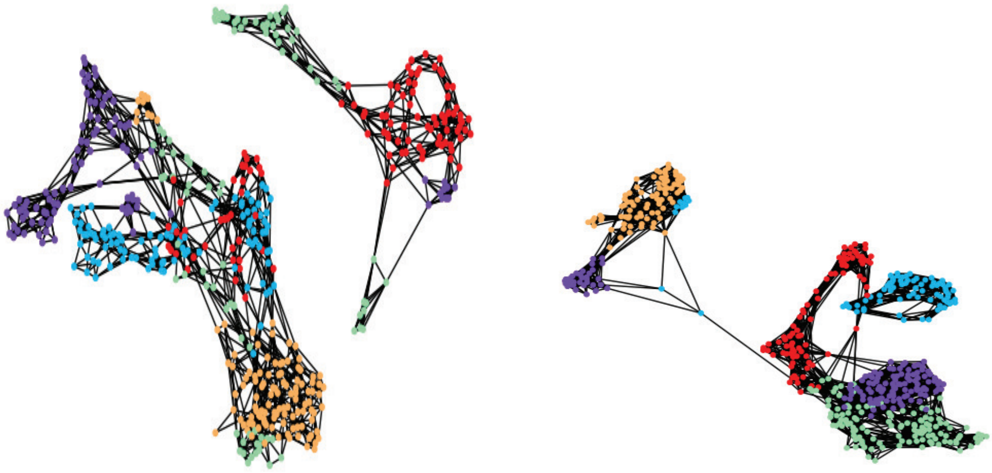

Figure 18. Results obtained using, the unconstrained binary optimization. On the left where maximum node degree was trimmed to 8, there is a clear noise introduces by noise inherent to the quantum devices. On the right maximum node degree was increased to 20, what significantly reduces noise in the obtained solutions.

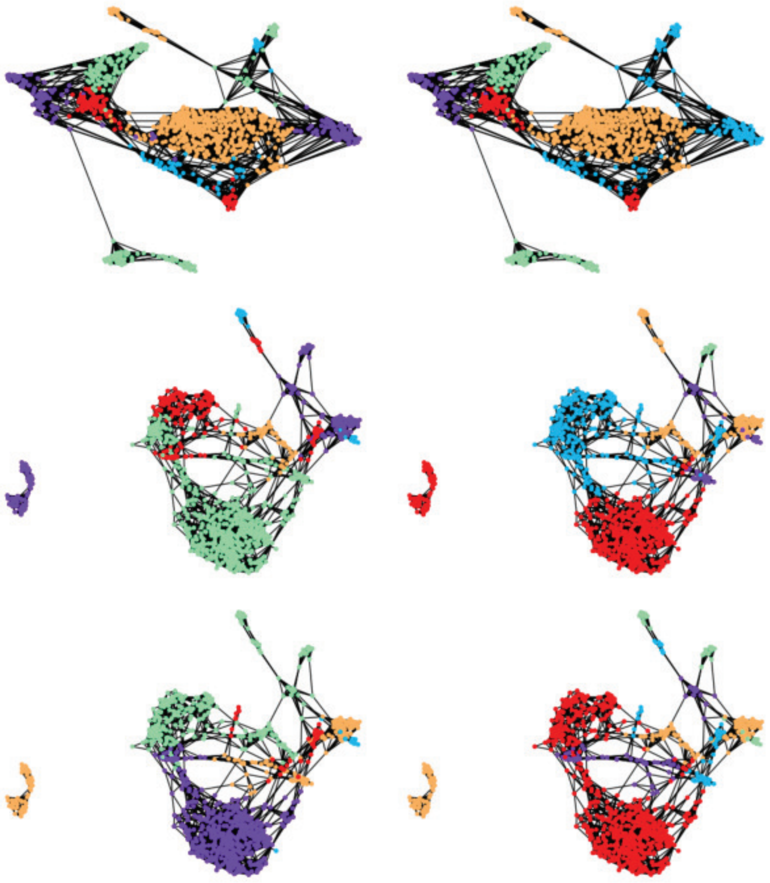

Figure 19. Graph partitions corresponding to the low energy states. First two graphs one the top were created with maximal node degree of 15. While four graphs below also had applied mutual nodes amplification prior to trimming.

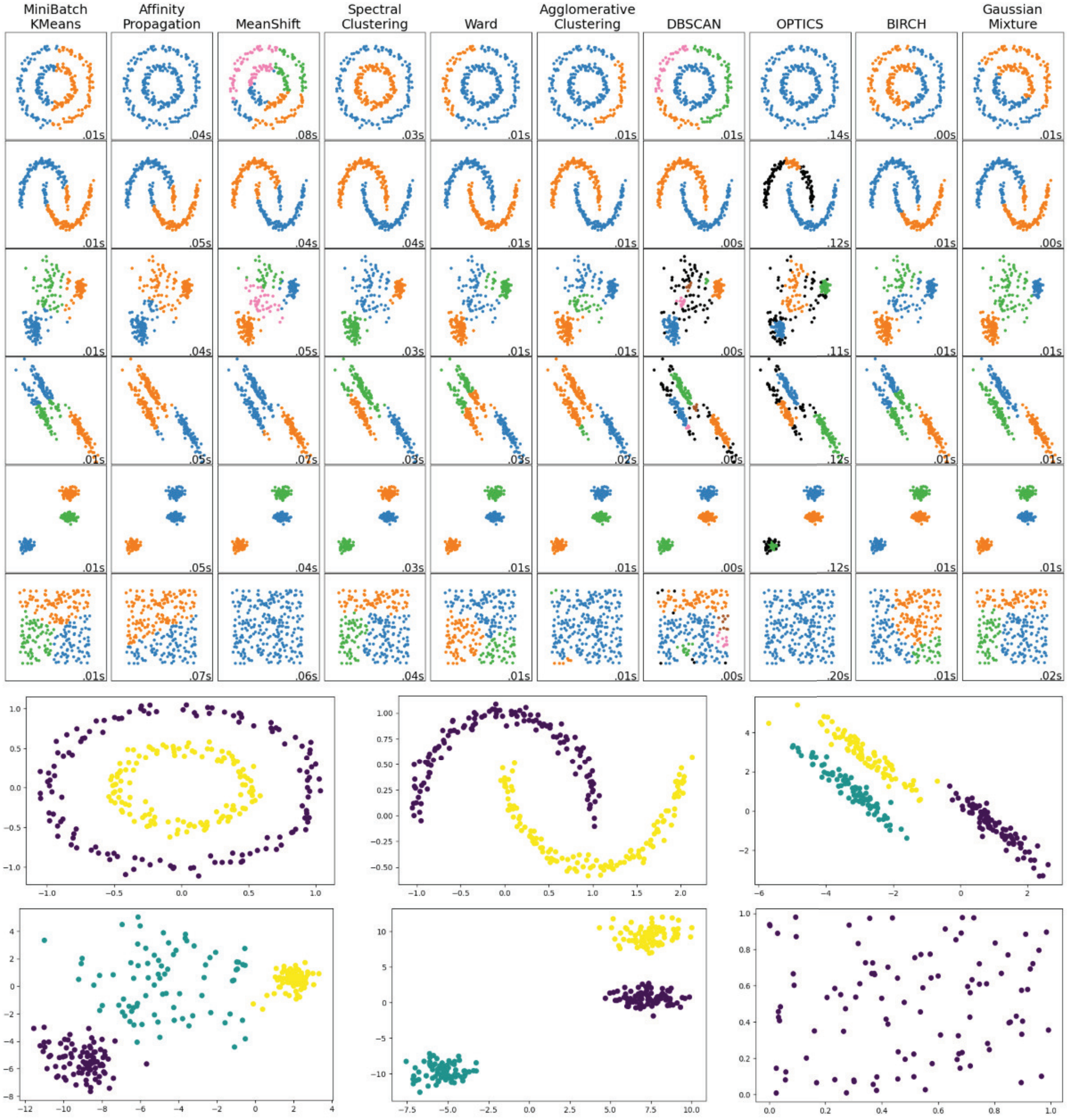

Figure 20. Comparison of QA clustering with other clustering methods. On the top - different classical clustering methods. On the bottom - results produced by quantum anneler. To define BQM, which can be solved by the quantum anneler, we generated SNN graph with 10 nearest neighbours.

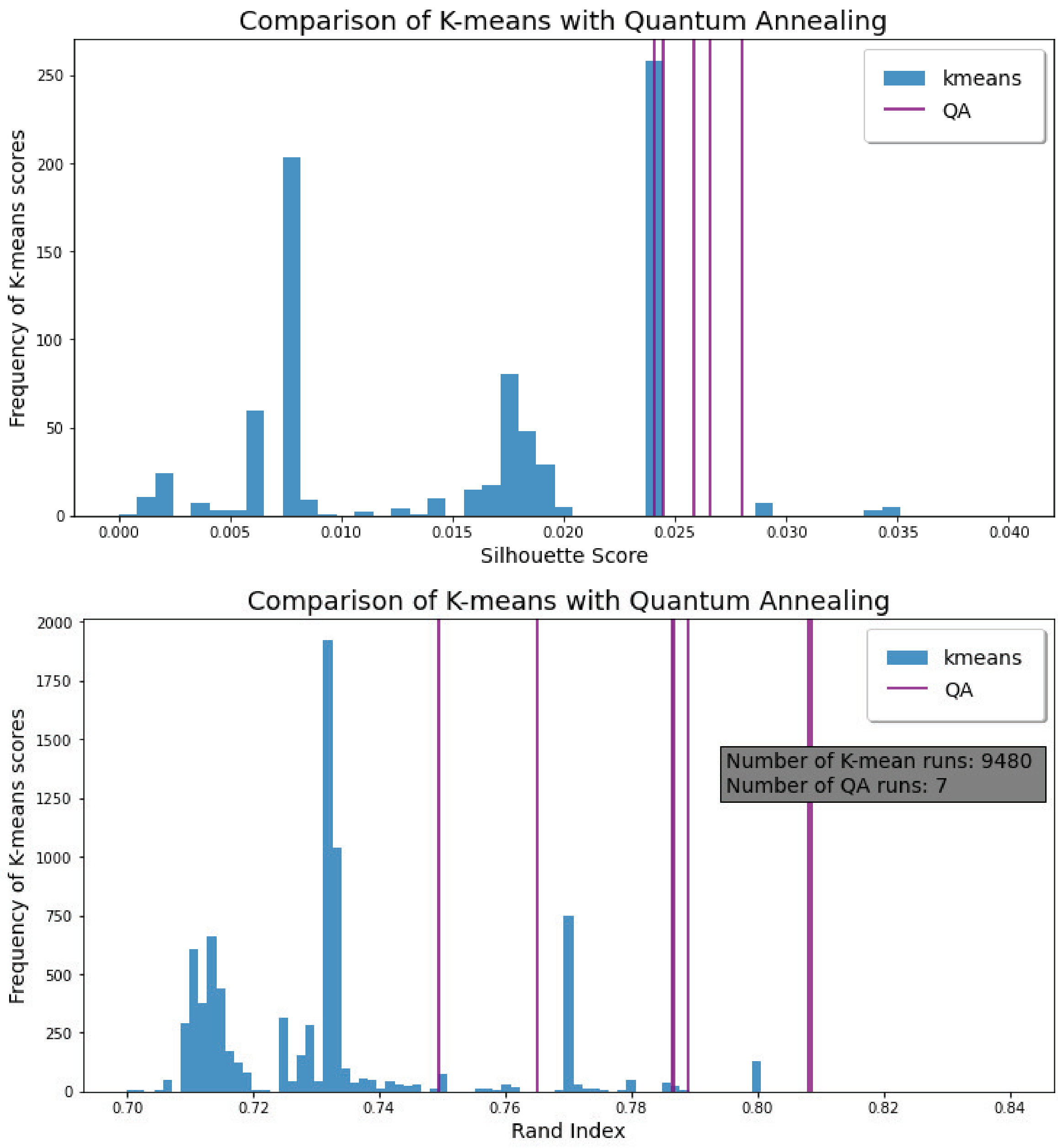

Figure 21. Comparing K-means and Quantum annealing-based clustering. Histograms (in blue) show distributions of Silhouette and Rand Index scores for the solutions found with the k-means algorithm. On the same graphs, the maroon vertical lines indicate analogous scores of the solutions sampled from the quantum annealer. It is apparent that the k-means algorithm tends to settle on certain local minima, which can be a limiting factor for data exploration and its conclusions. At the same time, the quantum annealing method samples solution space more holistically and finds solutions that surpass the k-means algorithm in both of the considered metrics.
